# The PreQuine Platform: A novel diagnostic tool for measuring glucose-6-phosphate dehydrogenase (G6PD) activity and hemoglobin concentration

**DOI:** 10.1371/journal.pone.0297918

**Published:** 2024-05-10

**Authors:** Robert Harper, Benedikt Ley, Md Alamgir Kabir, Graham Matulis, Lorenz von Seidlein, Mohammad Shafiul Alam, Bipin Adhikari, Bernard A. Okech, Alan L. Williams, Ric N. Price, Michael E. von Fricken

**Affiliations:** 1 In Vitro Diagnostic Solutions, Cherry Hill, New Jersey, United States of America; 2 Global and Tropical Health Division, Menzies School of Health Research and Charles Darwin University, Darwin, Australia; 3 Department of Environmental and Global Health, University of Florida, Gainesville, Florida, United States of America; 4 Faculty of Tropical Medicine, Mahidol-Oxford Tropical Medicine Research Unit, Mahidol University, Bangkok, Thailand; 5 Infectious Diseases Division, International Centre for Diarrheal Diseases Research, Bangladesh (icddr,b), Mohakhali, Dhaka, Bangladesh; 6 Department of Preventive Medicine and Biostatistics, F. Edward Herbert School of Medicine, Uniformed Services University of the Health Sciences, Bethesda, Maryland, United States of America; 7 Department of Family Medicine, Uniformed Services University of the Health Sciences, Bethesda, Maryland, United States of America; 8 Nuffield Department of Clinical Medicine, Centre for Tropical Medicine and Global Health, University of Oxford, Oxford, United Kingdom; 9 One Health Center of Excellence, University of Florida, Gainesville, Florida, United States of America; Shoklo Malaria Research Unit, THAILAND

## Abstract

Quantitative diagnosis of glucose-6-phosphate dehydrogenase (G6PD) deficiency is essential for the safe administration of 8-aminoquinoline based radical cure for the treatment of *Plasmodium vivax* infections. Here, we present the PreQuine Platform (IVDS, USA), a quantitative biosensor that uses a dual-analyte assay for the simultaneous measurement of Hemoglobin (Hgb) levels and G6PD enzyme activity within the same sample. The platform relies on a downloadable mobile application. The device requires 10μl of whole blood and works with a reflectance-based meter. Comparing the G6PD measurement normalized by Hgb of 12 samples from the PreQuine Platform with reference measurements methods (spectrophotometry, Pointe Scientific, USA and hemoglobin meter, HemoCue, Sweden) showed a positive and significant agreement with a slope of 1.0091 and an intercept of -0.0379 under laboratory conditions. Next steps will be to conduct field trials in Bangladesh, Cambodia, and the USA to assess diagnostic performance, user friendliness and acceptance.

## Introduction

*Plasmodium vivax* causes between 5–14 million malaria cases each year, with over two billion people at risk of infection [1,2], and is the predominant cause of malaria in areas outside of the African continent. In contrast to other malaria parasites, *P*. *vivax* (and *P*. *ovale*) can form dormant liver stages (hypnozoites) which reactivate weeks to months after the initial infection accounting for up to 80% of all cases of vivax malaria in some areas [3].

Only one class of drugs with hypnozoitocidal properties is currently licensed. 8-aminoquinoline compounds (8AQ), namely primaquine (PQ) and tafenoquine (TQ), can clear hypnozoites, and in combination with a blood schizontocidal are referred to as radical cure [4–6]. While well tolerated in most patients, PQ and TQ can cause hemolysis in individuals with glucose-6-phosphate dehydrogenase (G6PD) deficiency, an enzymopathy that affects between 400 to 500 million people worldwide [7–9]. Since routine testing for G6PD deficiency is rarely available in many endemic settings, clinicians often do not prescribe hypnozoitocidal treatment due to concerns of drug induced acute hemolytic anemia in patients with unknown G6PD deficiency. Hence, there is a critical need to Identify G6PD deficiency in patients and monitor hemoglobin (Hgb) concentrations at point-of-care, to guide radical cure and support overall clinical management of patients infected with *P*. *vivax* [1]. Wider use of G6PD diagnostics for the safe administration of radical cure will be critical to reduce relapse and the transmission of vivax malaria in high-risk populations. Scaled availability of point-of-care G6PD diagnostic kits for rural tropical regions, which suffer the largest burden of vivax malaria, will protect individuals vulnerable to 8-aminoquinolone drug-induced adverse events and enable individuals with normal G6PD levels to receive curative treatment [10].

The PreQuine Platform is a novel, point-of-care diagnostic kit developed by In Vitro Diagnostics Solutions (IVDS, USA) for the simultaneous, quantitative determination of G6PD enzyme activity and Hgb concentration. We report the first findings from the device when tested under laboratory conditions.

## Methods

### The PreQuine Platform

**Fig 1:**
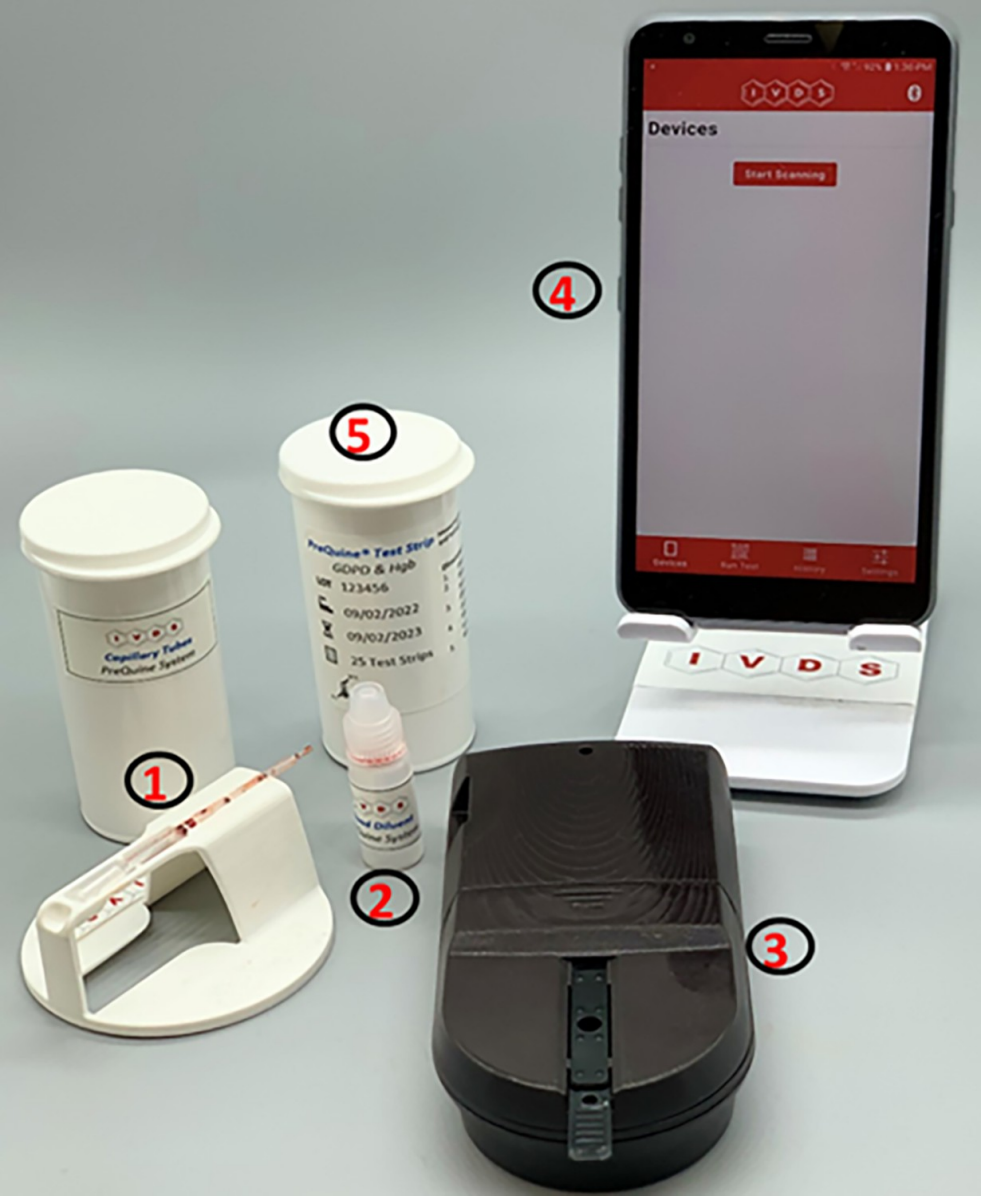
The PreQuine Platform consists of the following (1) Blood collection kits with single-use, fixed-volume pipettes, (2) Lysis buffer, (3) Handheld reflectance-based meter, (4) Cell phone application, and (5) Test strips for G6PD and Hgb detection.

The PreQuine Platform consists of a portable reflectometer, dual-analyte test strips, blood collection kits, fixed-volume, single-use pipettes, lysing buffer, and a mobile application (Fig 1). Each lot of test strips is validated by scanning a QR code which contains information concerning the lot number, expiration date, and calibration constants needed for calculating Hgb and G6PD levels. A total of 10 μl of capillary or venous blood is added to the lysis buffer using a single-use pipette, and the resulting suspension is mixed thoroughly. Using a second pipette, 20 μl of the blood-buffer suspension are then added to a test strip inserted into the meter cassette guide. A precise formulation of enzymes, bioactive components, surfactants, and excipients immobilized within the test strip membranes produce a colorimetric signal proportional to analyte levels in the sample. The reflectometer detects and transmits the colorimetric data to the mobile app for display, storage, and dissemination. The mobile app also displays step-by-step directions of use. A normalized G6PD measurement is displayed within 6 minutes, the measurement of Hgb concentration is done within 1 minute.

### Dual analyte test strip design

**Fig 2:**
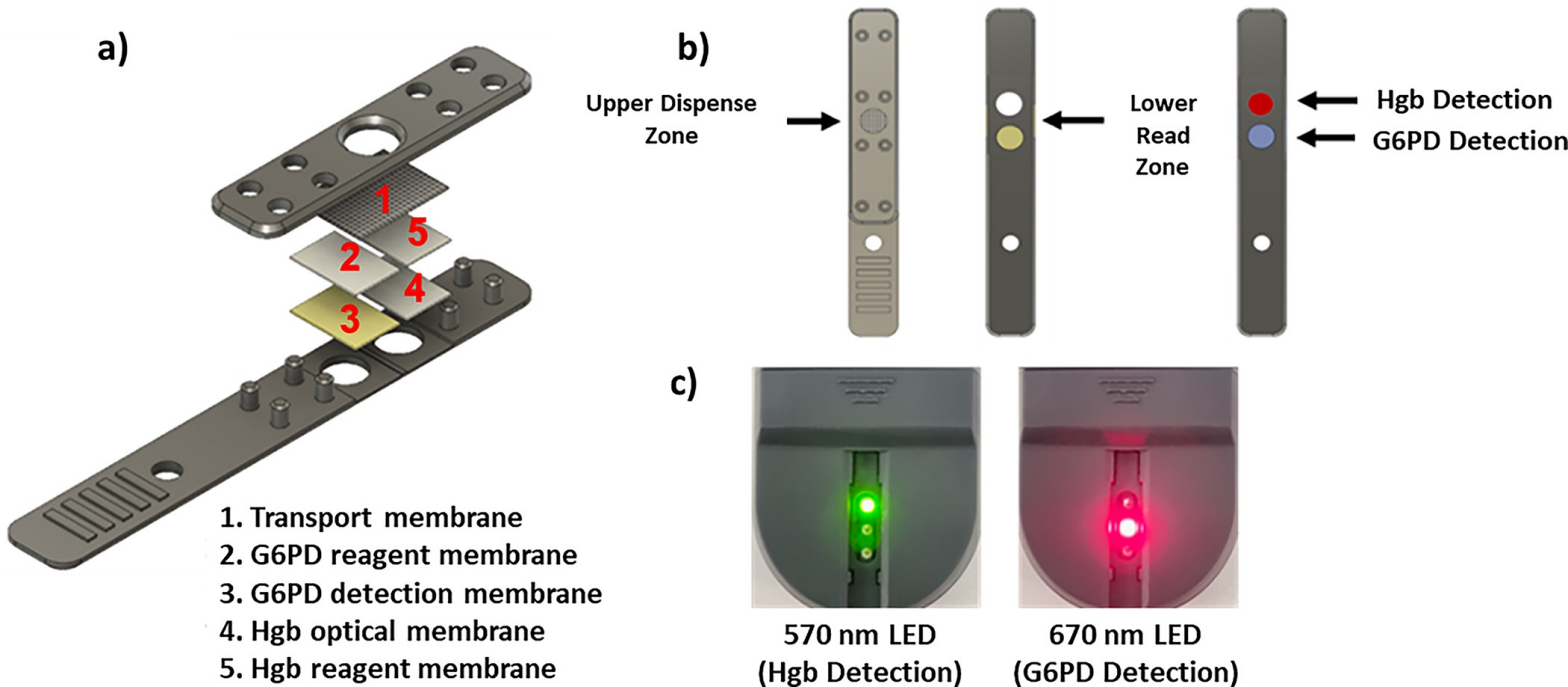
a) Exploded view of the G6PD dual analyte test strip. (1) a spreading layer (transport membrane), (2) a G6PD reagent membrane (hollow fiber membrane with electron mediator, inhibitor, and substrate), (3) a G6PD detection membrane (mixed mesh < 1 μm) facing the LED, (4) a reagent membrane (polyether sulfone membrane containing sodium azide and sodium nitrite) and (5) an optical membrane (polyether sulfone membrane < 1 μm) facing the LED. b) Top and bottom view of the fully assembled test strip with representative color change after test strip chemical reaction (far right). c) PreQuine Platform’s reflectance-based meter shows both 570 and 670 nm LEDs that are used for the simultaneous detection of Hgb and G6PD, respectively.

The design schema of the dual analyte test strip for simultaneous detection of Hgb concentration and G6PD enzyme activity is shown in Fig 2. A hydrophilic spreading layer (transport membrane), which contains a chelating agent, helps to filter cell debris while evenly distributing the sample laterally across the underlying membrane layers. The applied sample then migrates vertically through the Hgb and G6PD reaction layers, eventually reaching the read zones positioned over the 570nm (for Hgb detection) and 670nm (for G6PD detection) LEDs, which are embedded in the reflectometer.

### G6PD detection mechanism

**Fig 3:**
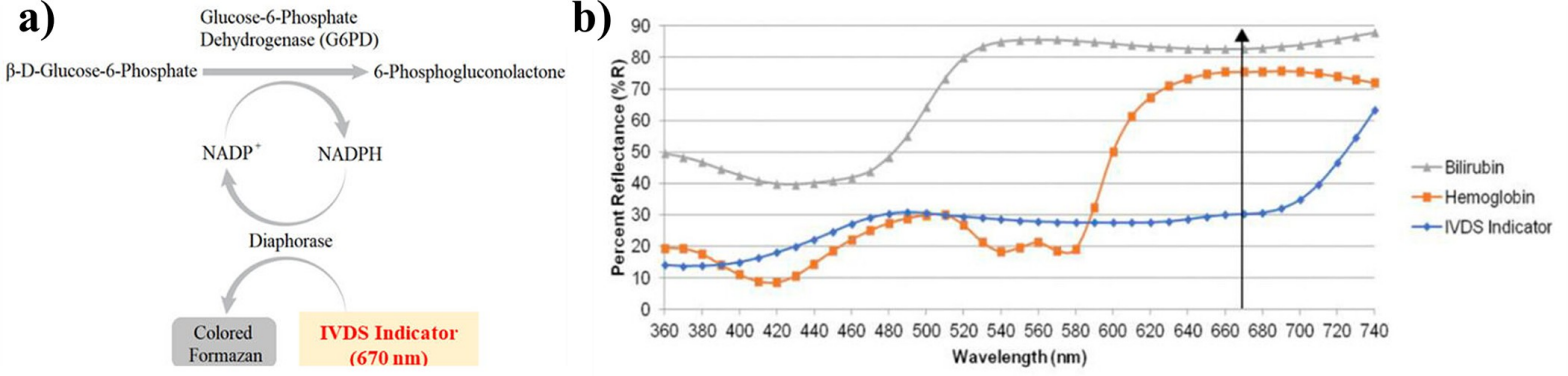
a) G6PD assay reaction mechanism. b) PreQuine Platform’s unique indicator showing free of interference with Bilirubin and Hemoglobin.

The reaction mechanism for the G6PD measurement is shown in Fig 3A. The chemistry utilizes a substrate β-D-glucose-6-phosphate disodium salt (G6PD substrate), NADP+, diaphorase (electron mediator), and a tetrazolium salt, which eventually produce formazan, a colored end-reaction product. The chosen formazan reaction product is outside of the spectral wavelength maxima of hemoglobin and bilirubin, reducing the potential for assay interference from these two compounds (Fig 3B). Maleimide is also added to the membrane as a sequestering compound to reduce the creation of secondary NADPH resulting from reactions with 6-phosphogluconate dehydrogenase. The PreQuine Platform incorporates a Temperature Correction Factor (TCF) for G6PD activity measurements, similar to other G6PD point-of-care quantitative devices [11], which is critical given that the G6PD enzyme activity is temperature dependent.

### Hgb detection mechanism

**Fig 4:**
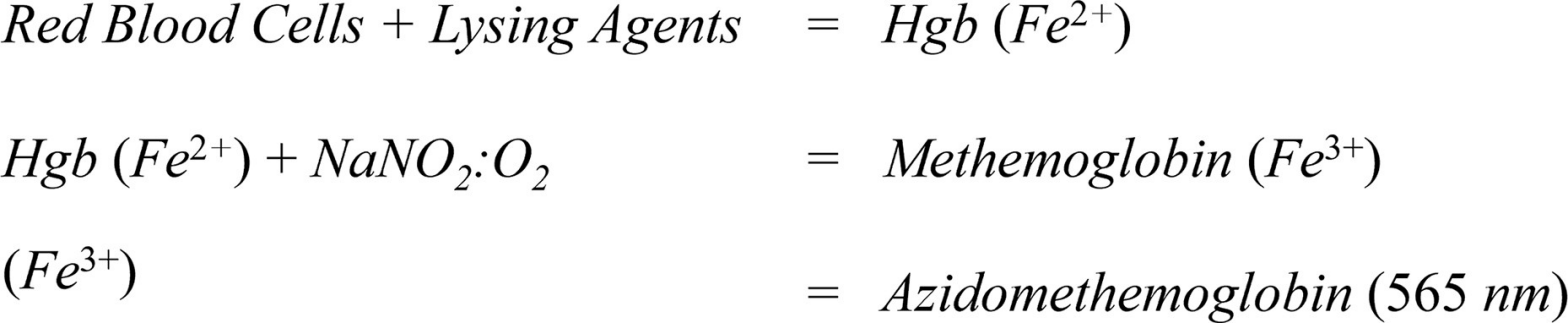
Hgb assay reaction mechanism.

The reaction mechanism for Hgb detection is shown in Fig 4. Free Hgb is liberated from the blood sample by mixing with the lysing buffer. After traversing the transport membrane, the sample flows through a polyether sulfone (PES) membrane coated with an oxidizer and sodium azide to convert the free Hgb to azide methemoglobin (Fig 2A, layer 5). The optical membrane is an anisotropic PES membrane facing the LED, which collects the colored reaction product and serves as the Hgb read zone.

### Preparation of blood samples

A total of 10 ml of deidentified healthy donor venous blood (heparinized) were purchased from BioIVT (USA) and aliquoted. The native G6PD enzyme activity was assayed by spectrophotometry on a temperature-controlled Genesys 10S (Thermo Scientific, Waltham, MA) at 37°C using commercially available reagents (Cat# G7583180, Pointe Scientific, Canton, MI) and the Hgb value of the blood was determined using the HemoCue HB 201 System (HemoCue, Angelholm, Sweden). G6PD enzyme activity was adjusted via freeze/thaw cycles and/or spiking with recombinant G6PD enzyme (Cat# 078A0020, Calzyme, San Luis Obispo, CA) while Hgb levels were adjusted through the addition and subtraction of plasma. This generated blood samples with G6PD enzyme activity ranging from 0–300 U/dL and with Hgb values ranging from 0–25 g/dL. Prepared samples were assayed in triplicate using the PreQuine Platform and the average values were used to generate a best-fit polynomial equation relating reflectance (%R) to Hgb (g/dL) or G6PD (U/dL). These polynomial equations were used to calculate G6PD and Hgb levels from the %R data. The calculated values were then compared to measurements obtained using reference assays (spectrophotometry with Pointe Scientific reagents and HB 201 HemoCue).

### Simultaneous G6PD and Hgb detection

G6PD enzyme activity must be normalized by Hgb concentration to provide a meaningful reading. To evaluate the performance of the PreQuine assay, we encoded the polynomial equations relating %R to G6PD (U/dL) and %R to Hgb (g/dL) into the QR code that is scanned using the PreQuine mobile app (the QR code also contains lot information and expiration date of the test strips) and programmed the results output to be expressed in normalized values of G6PD activity (U/g Hgb). We then obtained twelve de-identified blood samples with varying levels of G6PD enzyme activity (provided by PATH, USA, as G6PD references). The samples were assayed on the PreQuine Platform, HB 201, and using spectrophotometry to compare the G6PD enzyme activity values as determined by the PreQuine Platform with the values obtained from the reference assays.

## Results

### Performance of PreQuine system for simultaneous G6PD and Hgb measurement

**Fig 5:**
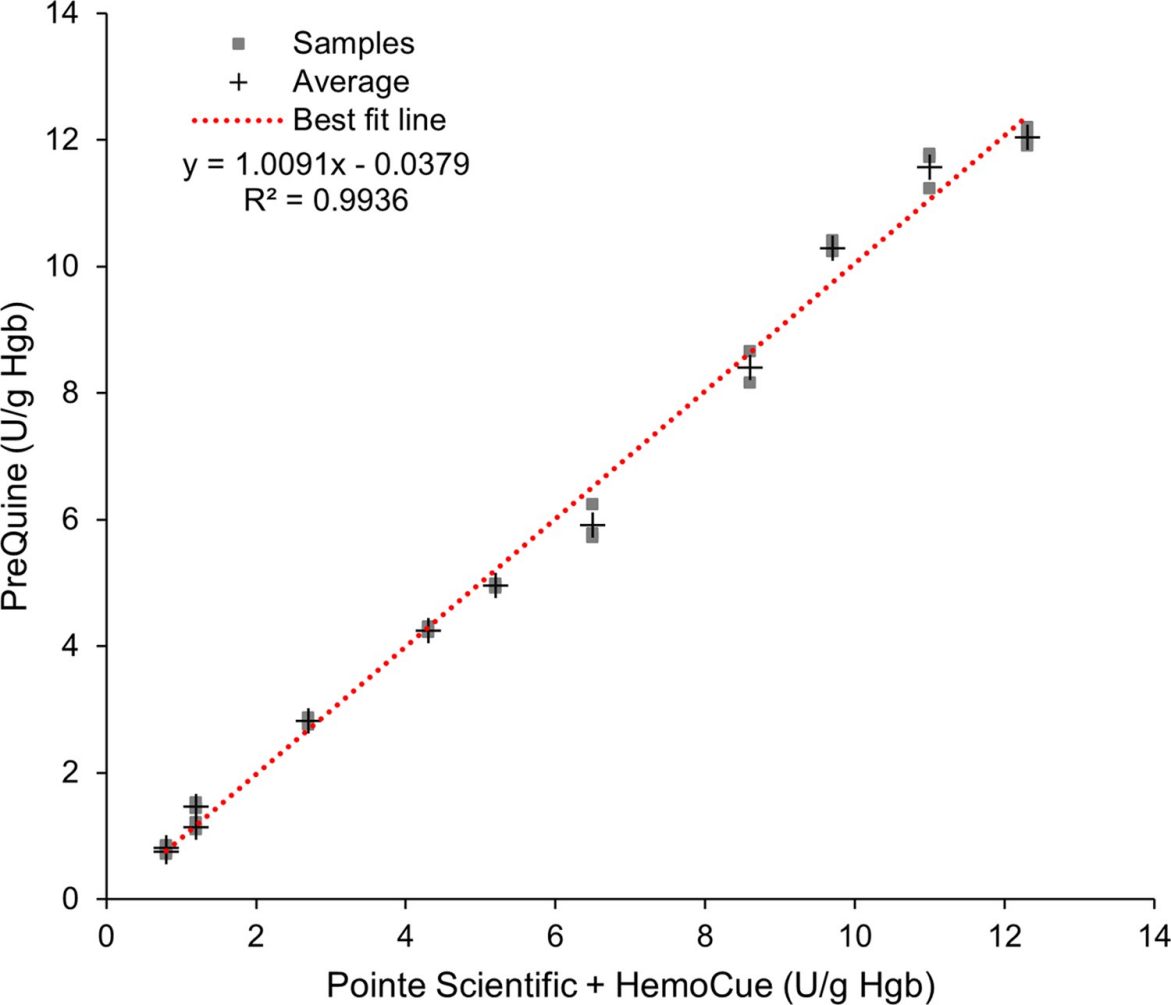
The performance comparison of the PreQuine Platform to measure the activity of the G6PD enzyme compared with the Pointe Scientific method measured by the spectrophotometer (Pointe Scientific reagents) in U/g Hgb.

Twelve de-identified blood samples with varying G6PD enzyme activities were assayed simultaneously using the HemoCue HB 201, Pointe Scientific reagents, and the PreQuine system. The linear relationship between the PreQuine Platform and combined HB 201/Pointe Scientific assays is shown in Fig 5. The PreQuine Platform showed a positive and significant linear agreement with the reference assays with a slope of 1.0091, an intercept of -0.0379, and R^2^ of 0.9936.

### Dose response of G6PD enzyme activity using PreQuine platform

**Fig 6:**
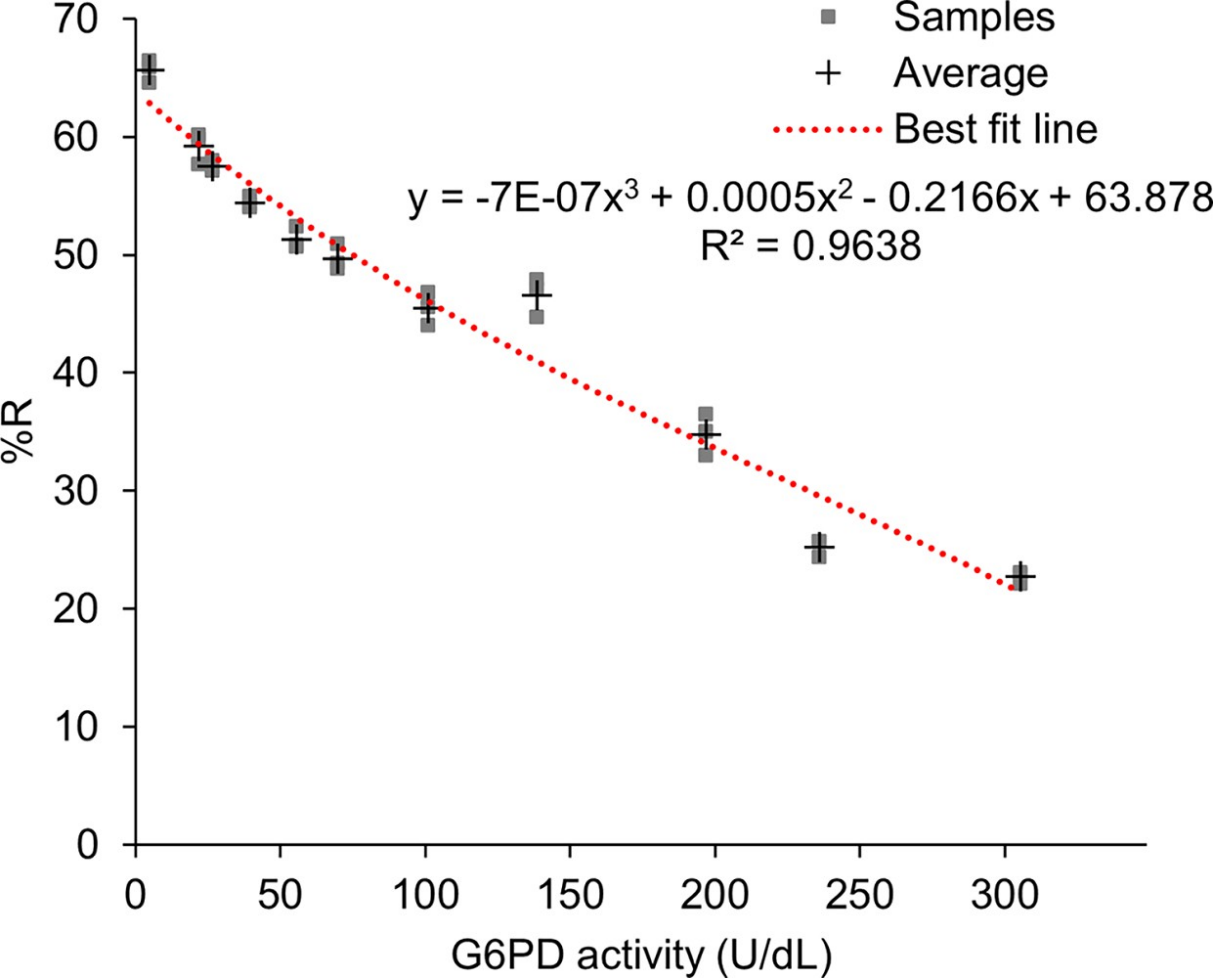
3rd order polynomial relationship between known values of G6PD/mL and percent reflectance values (derived from PreQuine G6PD assay) for G6PD-adjusted blood samples.

**Fig 7:**
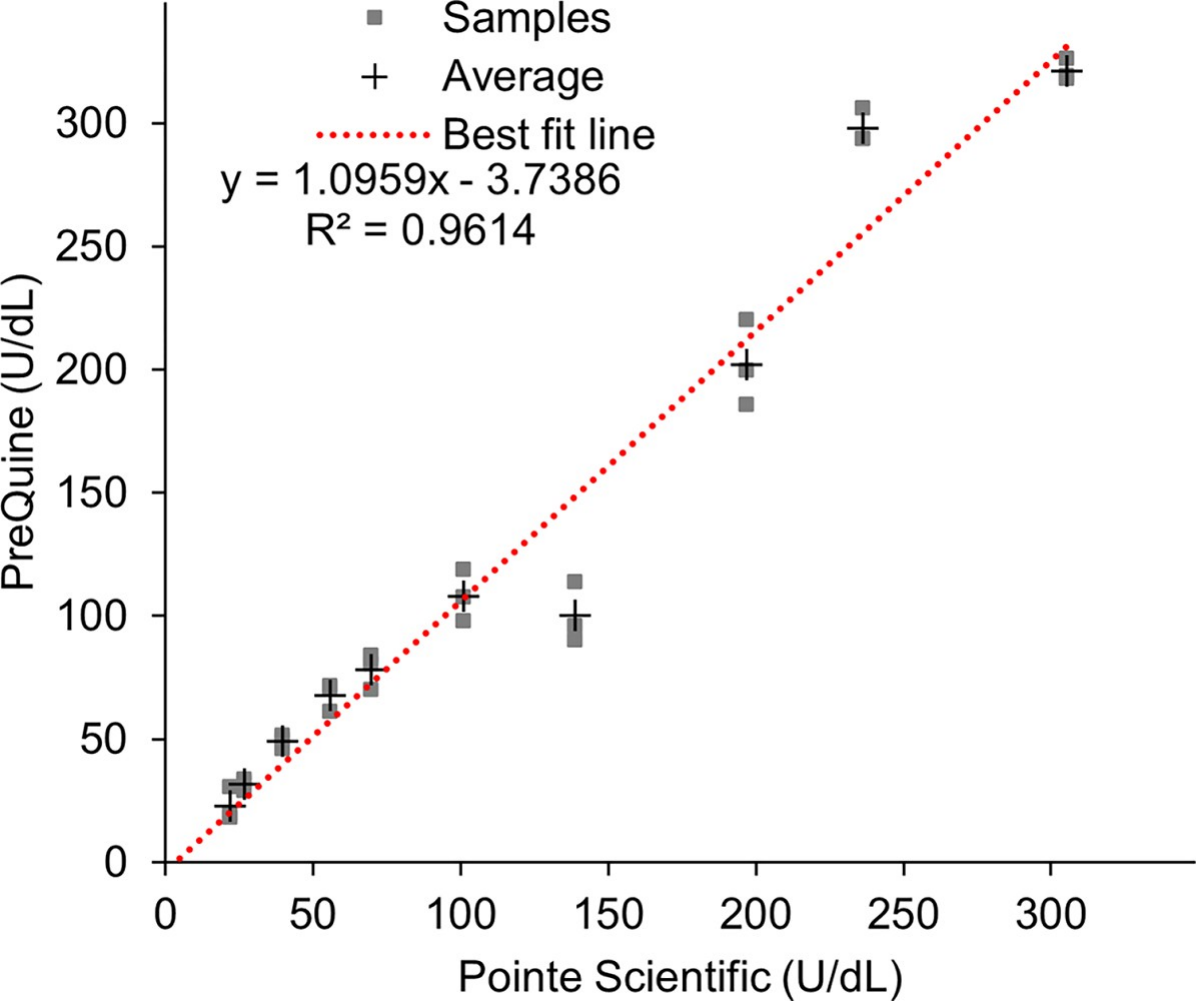
The first order linear regression relationship between U/dL values as measured by the Spectrophotometer (Pointe Scientifique) and as measured and derived by the PreQuine G6PD assay.

Fig 6 shows the dose response in %R of samples across the analytical range of 0–300 U/dL G6PD activity. Regression analysis revealed that a cubic fit polynomial described the dose response with R^2^ = 0.9638 and an average coefficient of variation of 2% (range 1–5%). The linear regression comparing the PreQuine Platform with the reference assay is shown in Fig 7. The PreQuine Platform showed a positive and significant linear agreement with spectrophotometry, with a slope of 1.0959, an intercept of -3.7386, and R^2^ of 0.9614.

### Dose response of Hgb using PreQuine platform

**Fig 8:**
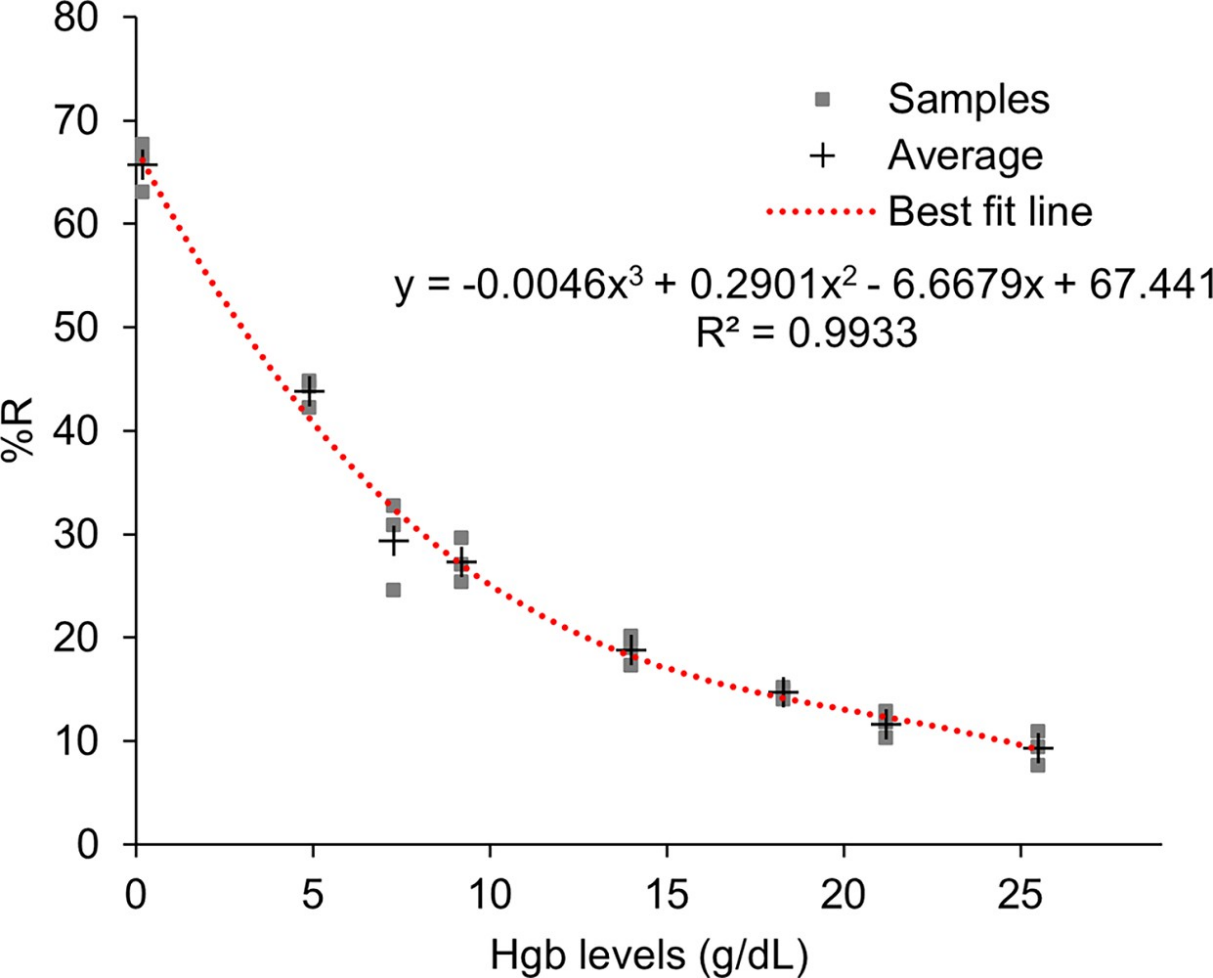
3rd order polynomial relationship between g/dL Hgb values (derived from HB 201) and percent reflectance values (derived from PreQuine Hgb assay) for Hgb-adjusted whole blood samples.

**Fig 9:**
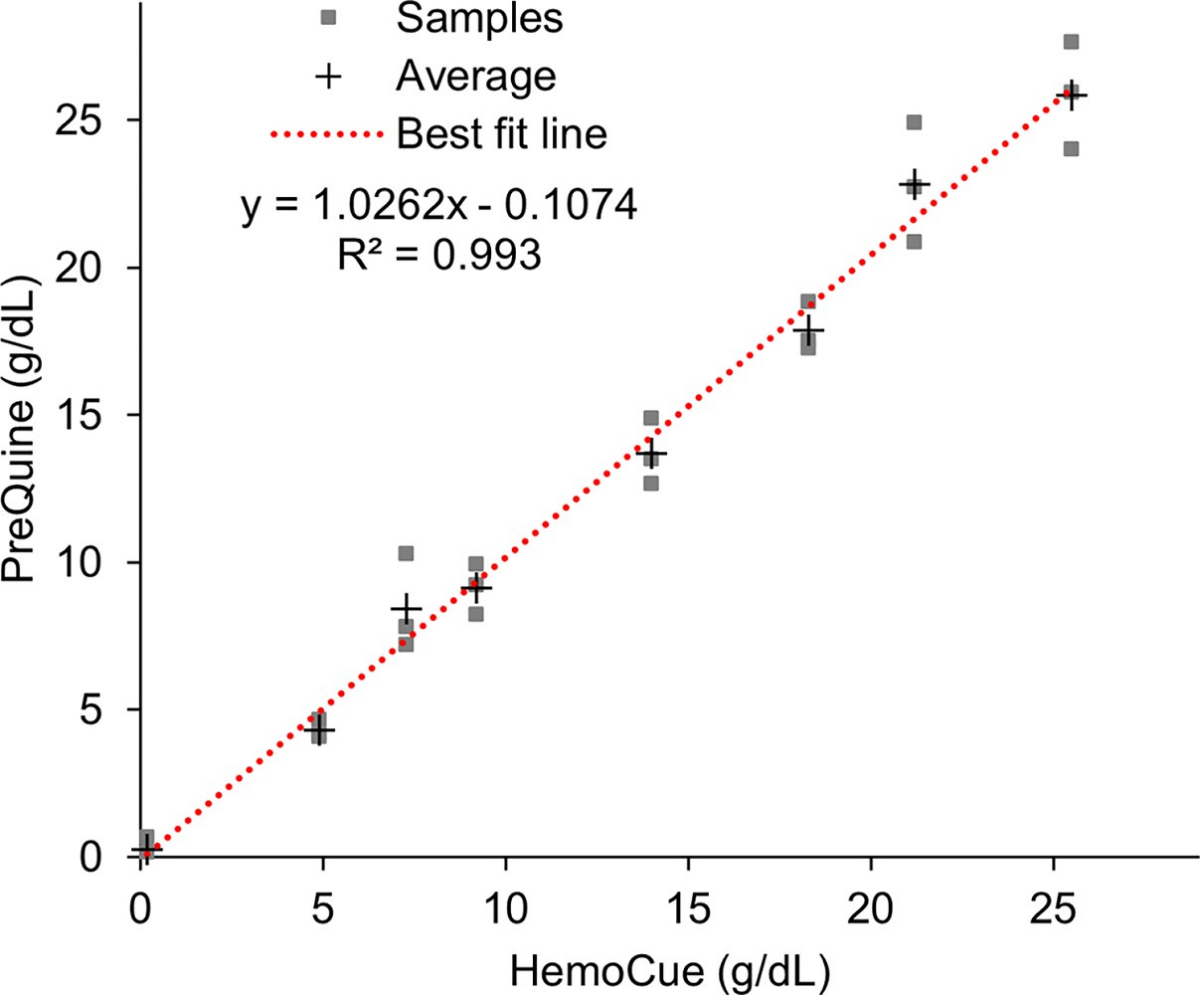
The first order linear regression relationship between g/dL values as measured by the HB 201 system and as measured and derived by the PreQuine Hgb system.

Fig 8 shows the dose response in %R of samples across the analytical range of 0–25 g/dL Hgb. Regression analysis revealed that a cubic fit polynomial described the dose response with R^2^ = 0.9933 and an average coefficient of variation of 9% (range 3–18%). The linear regression comparing the PreQuine Platform with HB 201 is shown in Fig 9. The PreQuine Platform showed a positive and significant linear agreement with the HB 201, with a slope of 1.0262, an intercept of -0.1074, and R^2^ of 0.993.

## Discussion

We found the PreQuine system to provide accurate measurements of G6PD activity levels up to 300 U/dL and Hgb values up to 25 g/dL, and a good correlation based on R^2^ with the corresponding reference methods, but further analysis of a larger sample size is required to determine statistically significant association (spectrophotometry, Pointe Scientific, USA; hemoglobin meter, HemoCue, Sweden). The PreQuine system is quantitative, does not require laboratory equipment, does not require cold chain for distribution or storage, and should remain functional at ambient tropical temperatures, but requires field validation to determine actual performance in vivo which will help us understand the impact of other environmental factors like humidity.

The dual-analyte design omits the need to run two assays in parallel and removes the need for health care providers to calculate G6PD activity. In contrast to other comparable devices the Prequine measures product formation from G6PD activity at 670nm and thereby avoids inference from the Hgb present in the blood sample, where Fe^2+^ from Hgb can yield the same color signal as the end reaction of the G6PD assay, resulting in an unwanted background signal. Furthermore, the chosen chemical indicator for G6PD detection has an absorbance maximum outside of the absorbance range of bilirubin.

The PreQuine Platform relies on a mobile application that connects the meter device via Bluetooth to a mobile phone. This allows users to store patient ID, test results, and geolocation information, while also allowing for the calculation of normalized G6PD activity in U/g Hgb. This approach reduces the hardware costs for the end user, simplifies software updates worldwide, and provides a real time, step-by-step guide to the user while performing a measurement. The app also provides a platform for additional features that can be added on later such as telehealth consultation, and if requested by the user uploading of test results to centralized databases. While various assays have been developed to determine G6PD deficiency, many of have limited utility in malaria-endemic regions, including: the need for expensive laboratory equipment, cold chain requirements, the need of a separate assay for Hgb quantification, cost, and inadequate assay sensitivities and specificities [12].

The reported results were performed by trained scientists in a laboratory setting, thus far the PreQuine Platform has not been tested under field conditions on samples from malaria patients. It is expected that the observed correlation may differ under more realistic conditions. To assess field feasibility, the PreQuine Platform will be trialed at field sites in Bangladesh, Cambodia, and the United States following standard procedures [13].

## Conclusions

Initial laboratory-based data suggest that the PreQuine device shows good correlation across the physiological range of 0–300 U/dL G6PD activity and 0–25 g/dL Hgb, when compared to the respective reference assays. Field studies will be critical to validate these findings, assess the overall device performance, monitor user friendliness and acceptance, and eventually examine PreQuine performance in comparison to other commercially available point-of-care G6PD devices.
